# Burden, access, and disparities in kidney disease

**DOI:** 10.1590/1414-431X20198338

**Published:** 2019-03-25

**Authors:** D.C. Crews, A.K. Bello, G. Saadi, World Kidney Day Steering Committee

**Affiliations:** 1 Division of Nephrology, Department of Medicine, Johns Hopkins University School of Medicine, Baltimore, Maryland, USA; 2 Welch Center for Prevention, Epidemiology and Clinical Research, Johns Hopkins Medical Institutions, Baltimore, Maryland, USA; 3 Johns Hopkins Center for Health Equity, Johns Hopkins Medical Institutions, Baltimore, Maryland, USA; 4 Division of Nephrology & Transplant Immunology, Department of Medicine, University of Alberta, Edmonton, Canada; 5 Nephrology Unit, Department of Internal Medicine, Faculty of Medicine, Cairo University, Giza, Egypt

**Keywords:** Acute kidney injury, End stage renal disease, Global health, Health equity, Social determinants of health

## Abstract

This article was published in Kidney International volume 95, pages 242–248, https://doi.org/10.1016/j.kint.2018.11.007, Copyright World Kidney Day 2019 Steering Committee (2019) and is reprinted concurrently in several journals. The articles cover identical concepts and wording, but vary in minor stylistic and spelling changes, detail, and length of manuscript in keeping with each journal's style. Any of these versions may be used in citing this article. Note that all authors contributed equally to the conception, preparation, and editing of the manuscript.

Kidney disease is a global public health problem, affecting over 750 million persons worldwide. The burden of kidney disease varies substantially across the world, as does its detection and treatment. In many settings, rates of kidney disease and the provision of its care are defined by socio-economic, cultural, and political factors leading to significant disparities. World Kidney Day 2019 offers an opportunity to raise awareness of kidney disease and highlight disparities in its burden and current state of global capacity for prevention and management. Here, we highlight that many countries still lack access to basic diagnostics, a trained nephrology workforce, universal access to primary health care, and renal replacement therapies. We point to the need for strengthening basic infrastructure for kidney care services for early detection and management of acute kidney injury and chronic kidney disease across all countries and advocate for more pragmatic approaches to providing renal replacement therapies. Achieving universal health coverage worldwide by 2030 is one of the World Health Organization's Sustainable Development Goals. While universal health coverage may not include all elements of kidney care in all countries, understanding what is feasible and important for a country or region with a focus on reducing the burden and consequences of kidney disease would be an important step towards achieving kidney health equity.

## Introduction

Kidney disease is a global public health problem that affects more than 750 million persons worldwide (1). The burden of kidney disease varies substantially across the world, as does its detection and treatment. Although the magnitude and impact of kidney disease is better defined in developed countries, emerging evidence suggests that developing countries have a similar or even greater kidney disease burden (2).

In many settings, rates of kidney disease and the provision of its care are defined by socioeconomic, cultural, and political factors, leading to significant disparities in disease burden, even in developed countries (3). These disparities exist across the spectrum of kidney disease — from preventive efforts to curb development of acute kidney injury (AKI) or chronic kidney disease (CKD), to screening for kidney disease among persons at high risk, to access to subspecialty care and treatment of kidney failure with renal replacement therapy (RRT). World Kidney Day 2019 offers an opportunity to raise awareness of kidney disease and highlight disparities in its burden and current state of global capacity for prevention and management. In this editorial, we highlight these disparities and emphasize the role of public policies and organizational structures in addressing them. We outline opportunities to improve our understanding of disparities in kidney disease, the best ways for them to be addressed, and how to streamline efforts toward achieving kidney health equity across the globe.

## Burden of kidney disease

Availability of data reflecting the full burden of kidney disease varies substantially because of limited or inconsistent data collection and surveillance practices worldwide (Table 1) (4). Whereas several countries have national data collection systems, particularly for end-stage renal disease (ESRD) (e.g., United States Renal Data System, Latin American Dialysis and Renal Transplant Registry, and Australia and New Zealand Dialysis and Transplant Registry), high-quality data regarding non-dialysis CKD is limited, and often the quality of ESRD data is quite variable across settings. This situation is of particular concern in low-income countries. For example, a meta-analysis of 90 studies on CKD burden conducted across Africa showed very few studies (only 3%) with robust data (5). The provision of adequate resources and a workforce to establish and maintain surveillance systems (e.g., screening programs and registries) is essential and requires substantial investment (6). Incorporating kidney disease surveillance parameters in existing chronic disease prevention programs might enhance global efforts toward obtaining high-quality information on kidney disease burden and attendant consequences.

**Table 1:** World Bank country group for chronic kidney disease gaps.

CKD care	Low-income countries (%)	Lower-middle-income countries (%)	Upper-middle-income countries (%)	High-income countries (%)
Governmental recognition of CKD as a health priority	59	50	17	29
Government funds all aspects of CKD care	13	21	40	53
Availability of CKD management and referral guidelines (international, national, or regional)	46	73	83	97
Existence of current CKD detection programs	6	24	24	32
Availability of dialysis registries	24	48	72	89
Availability of academic centers for renal clinical trial management	12	34	62	63

CKD: chronic kidney disease. Data from Bello et al. 4.

In addition to a need for functional surveillance systems, the global importance of kidney disease (including AKI and CKD) is yet to be widely acknowledged, making it a neglected disease on the global policy agenda. For instance, the World Health Organization (WHO) Global Action Plan for the Prevention and Control of Non-Communicable Diseases (NCDs) (2013) focuses on cardiovascular diseases, cancer, chronic respiratory diseases, and diabetes but not kidney disease, despite advocacy efforts by relevant stakeholders such as the International Society of Nephrology and the International Federation of Kidney Foundations through activities such as World Kidney Day. This situation is quite concerning because estimates from the Global Burden of Disease study in 2015 showed that around 1.2 million people were known to have died of CKD (7), and more than 2 million people died in 2010 because they had no access to dialysis. It is estimated that another 1.7 million die from AKI on an annual basis (8,9). It is possible, therefore, that kidney disease may contribute to more deaths than the 4 main NCDs targeted by the current NCD Action Plan.

### Risk factors for kidney disease

Data in recent decades have linked a host of genetic, environmental, sociodemographic, and clinical factors to risk of kidney disease. The population burden of kidney disease is known to correlate with socially defined factors in most societies across the world. This phenomenon is better documented in high-income countries, where racial/ethnic minority groups and people of low socioeconomic status carry a high burden of disease. Extensive data have demonstrated that racial and ethnic minorities (e.g., African Americans in the United States, Aboriginal groups in Canada and Australia, Indo-Asians in the United Kingdom, and others) are affected disproportionately by advanced and progressive kidney disease (10–12). The associations of socioeconomic status and risk of progressive CKD and eventual kidney failure also have been well described, with persons of lower socioeconomic status bearing the greatest burden (13,14).

Recent works have associated apolipoprotein L1 risk variants (15,16) with increased kidney disease burden among persons with African ancestry. In Central America and Southeastern Mexico, Mesoamerican nephropathy (also referred to as CKD of unknown causes) has emerged as an important cause of kidney disease. While multiple exposures have been studied for their potential role in CKD of unknown causes, recurrent dehydration and heat stress are common denominators in most cases (17). Other perhaps more readily modifiable risk factors for kidney disease and CKD progression that disproportionately affect socially disadvantaged groups also have been identified, including disparate rates and poor control of clinical risk factors such as diabetes and hypertension, as well as lifestyle behaviors.

Diabetes is the leading cause of advanced kidney disease worldwide (18). In 2016, 1 in 11 adults worldwide had diabetes and more than 80% were living in low- and middle-income countries (19) where resources for optimal care are limited. Hypertension is also estimated to affect 1 billion persons worldwide (20) and is the second leading attributed cause of CKD (18). Hypertension control is important for slowing CKD progression and decreasing mortality risk among persons with or without CKD. Hypertension is present in more than 90% of persons with advanced kidney disease (18), yet racial/ethnic minorities and low-income persons with CKD who live in high-income countries have poorer blood pressure control than their more socially advantaged counterparts (21).

Lifestyle behaviors, including dietary patterns, are strongly influenced by socioeconomic status. In recent years, several healthful dietary patterns have been associated with favorable CKD outcomes (22). Low-income persons often face barriers to healthful eating that may increase their risk of kidney disease (23–25 [Bibr B26]). People of low socioeconomic status often experience food insecurity (i.e., limited access to affordable nutritious foods), which is a risk factor for CKD26 and progression to kidney failure (27). In low-income countries, food insecurity may lead to undernutrition and starvation, which has implications for the individual and, in the case of women of child-bearing age, could lead to their children having low birth weight and related sequelae, including CKD (28). Rates of undernourishment are as high as 35% or more in countries such as Haiti, Namibia, and Zambia (29). However, in high-income countries, food insecurity is associated with overnutrition, and persons with food insecurity have increased risk of overweight and obesity (30,31). Further, food insecurity has been associated with several diet-related conditions, including diabetes and hypertension.

### Acute kidney injury

AKI is an underdetected condition that is estimated to occur in 8 to 16% of hospital admissions (32) and is now well established as a risk factor for CKD (33). Disparities in AKI risk are also common, following a pattern similar to that observed in persons with CKD (34). AKI related to nephrotoxins, alternative (traditional) medicines, infectious agents, and hospitalizations and related procedures are more pronounced in low-income and lower-middle-income countries and contribute to increased risk of mortality and CKD in those settings (35). Importantly, the majority of annual AKI cases worldwide (85% of more than 13 million cases) are experienced in low-income and lower-middle-income countries, leading to 1.4 million deaths (36).

## Health policies and financing of kidney disease care

Because of the complex and costly nature of kidney disease care, its provision is tightly linked with the public policies and financial status of individual countries. For example, gross domestic product is correlated with lower dialysis-to-transplantation ratios, suggesting greater rates of kidney transplantation in more financially solvent nations. In several high-income countries, universal health care is provided by the government and includes CKD and ESRD care. In other countries, such as the United States, ESRD care is publicly financed for citizens; however, optimal treatment of CKD and its risk factors may not be accessible for persons lacking health insurance, and regular care of undocumented immigrants with kidney disease is not covered (37). In low-income and lower-middle-income countries, neither CKD nor ESRD care may be publicly financed, and CKD prevention efforts are often limited. In several such countries, collaborations between public and private sectors have emerged to provide funding for RRT. For example, in Karachi, Pakistan, a program of dialysis and kidney transplantation through joint community and government funding has existed for more than 25 years (38).

In many settings, persons with advanced CKD who have no or limited public or private sector funding for care shoulder a substantial financial burden. A systematic review of 260 studies including patients from 30 countries identified significant challenges, including fragmented care of indeterminate duration, reliance on emergency care, and fear of catastrophic life events because of diminished financial capacity to withstand them (39). Authors of another study conducted in Mexico found that patients and families were burdened with having to navigate multiple health and social care structures, negotiate treatments and costs, finance their health care, and manage health information (40). Challenges may be even greater for families of children with ESRD, because many regions lack qualified pediatric care centers.

## Organization and structures for kidney disease care

The lack of recognition and therefore the absence of a global action plan for kidney disease partly explains the substantial variation in structures and capacity for kidney care around the globe. This situation has resulted in variations in government priorities, health care budgets, care structures, and human resource availability (41). Effective and sustainable advocacy efforts are needed at global, regional, and national levels to get kidney disease recognized and placed on the global policy agenda.

In 2017, the International Society of Nephrology collected data on country-level capacity for kidney care delivery using a survey, the Global Kidney Health Atlas (4), which aligned with the WHO's building blocks of a health system. The Global Kidney Health Atlas highlights limited awareness of kidney disease and its consequences and persistent inequities in resources required to tackle the burden of kidney disease across the globe. For example, CKD was recognized as a health care priority by government in only 36% of countries that participated in this survey. The priority was inversely related to income level: CKD was a health care priority in more than half of low-income and lower-middle-income countries but in less than 30% of upper-middle-income and high-income countries.

**Figure 1:**
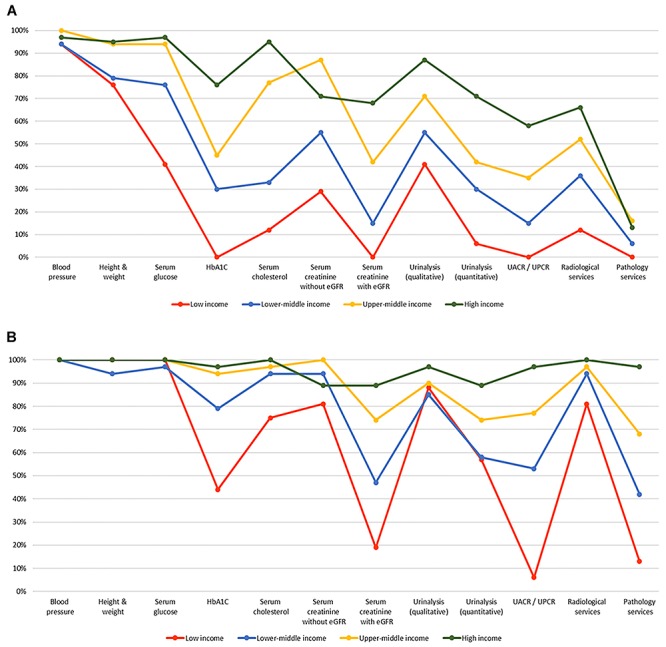
Health care services for identification and management of chronic kidney disease by country income level. (**A**) Primary care (i.e., basic health facilities at community levels [e.g., clinics, dispensaries, and small local hospitals]). (**B**) Secondary/specialty care (i.e., health facilities at a level higher than primary care [e.g., clinics, hospitals, and academic centers]). eGFR: estimated glomerular filtration rate; HbA1C: glycated hemoglobin; UACR: urine albumin-to-creatinine ratio; UPCR: urine protein-to-creatinine ratio. Data from Bello et al. (4) and Htay et al. (42)

Regarding capacity and resources for kidney care, many countries still lack access to basic diagnostics, a trained nephrology workforce, universal access to primary health care, and RRT technologies. Low-income and lower-middle-income countries, especially in Africa, had limited services for the diagnosis, management, and monitoring of CKD at the primary care level, with only 12% having serum creatinine measurement, including estimated glomerular filtration rate. Twenty-nine percent of low-income countries had access to qualitative urinalysis using urine test strips; however, no low-income country had access to urine albumin-to-creatinine ratio or urine protein-to-creatinine ratio measurements at the primary care level. Across all world countries, availability of services at the secondary/tertiary care level was considerably higher than at the primary care level (Figure 1A and B) (4,42).

### Renal replacement therapies

The distribution of RRT technologies varied widely. On the surface, all countries reported having long-term hemodialysis services, and more than 90% of countries reported having short-term hemodialysis services. However, access to and distribution of RRT across countries and regions was highly inequitable, often requiring prohibitive out-of-pocket expenditure, particularly in low-income regions. For instance, more than 90% of upper-middle-income and high-income countries reported having chronic peritoneal dialysis services, whereas these services were available in 64 and 35% of low-income and lower-middle-income countries, respectively. In comparison, acute peritoneal dialysis had the lowest availability across all countries. More than 90% of upper-middle-income and high-income countries reported having kidney transplant services, with more than 85% of these countries reporting both living and deceased donors as the organ source. As expected, low-income countries had the lowest availability of kidney transplant services, with only 12% reporting availability, and live donors as the only source.

### Workforce for kidney care

**Figure 2:**
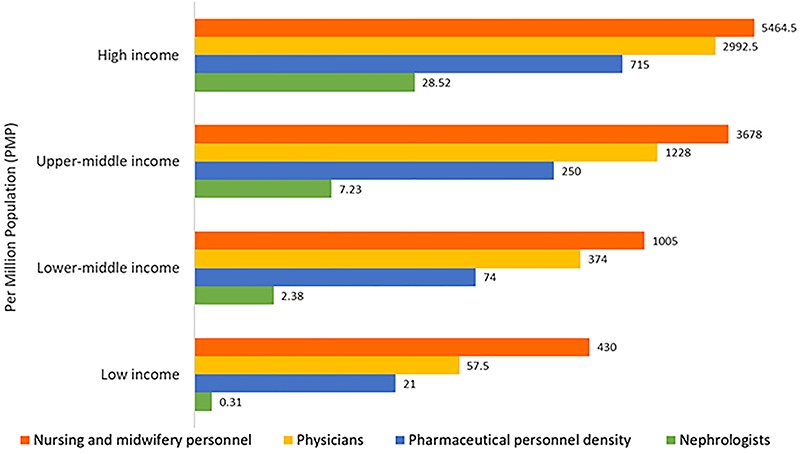
Nephrologist availability (density per million population) compared with physician, nursing, and pharmaceutical personnel availability by country income level. Pharmaceutical personnel include pharmacists, pharmaceutical assistants, and pharmaceutical technicians. Nursing and midwifery personnel include professional nurses, professional midwives, auxiliary nurses, auxiliary midwives, enrolled nurses, enrolled midwives, and related occupations such as dental nurses. A logarithmic scale was used for the x-axis [log(x+1)] because of the large range in provider density. Data from Bello et al. (4), Osman et al. (43), and the World Health Organization (for pharmaceutical personnel: http://apps.who.int/gho/data/view.main.PHARMS and http://apps.who.int/gho/data/node.main-amro.HWF?lang=en, for nursing and midwifery personnel: http://apps.who.int/gho/data/view.main.NURSES, for physicians: http://apps.who.int/gho/data/view.main.92000) (44).

Considerable international variation was also noted in the distribution of the kidney care workforce, particularly nephrologists. The lowest density (<5 nephrologists per million population) was very common in low-income countries, whereas the highest density (>15 nephrologists per million population) was reported mainly in high-income countries (Figure 2) (4,43,44). Most countries reported nephrologists as primarily responsible for both CKD and AKI care. Primary care physicians had more responsibility for CKD care than for AKI care, as 64% of countries reported that primary care physicians are primarily responsible for CKD care and 35% reported that they are responsible for AKI care. Intensive care specialists were primarily responsible for AKI in 75% of countries, likely because AKI is typically treated in hospitals. However, only 45% of low-income countries reported that intensive care specialists were primarily responsible for AKI, compared with 90% of high-income countries; this discrepancy may be due to a general shortage of intensive care specialists in low-income countries.

The appropriate number of nephrologists in a country depends on many factors, including need, priority, and resources, and as such, there is no global standard with respect to nephrologist density. Regardless, the demonstrated low density in low-income countries calls for concern as nephrologists are essential to provide leadership in kidney disease care, and a lack of nephrologists may result in adverse consequences for policy and practice. However, it is quite encouraging that the number of nephrologists and nephropathologists is rising in low-income and lower-middle-income countries, in part thanks to fellowship programs supported by international nephrology organizations (45). It is important to note that the role of a nephrologist may differ depending on how the health care system is structured. The density statistic merely represents the number of nephrologists per million population and provides no indication of the adequacy to meet the needs of the population or quality of care, which depends on volume of patients with kidney disease and other workforce support (e.g., availability of multidisciplinary teams).

For other care providers essential for kidney care, international variations exist in distribution (availability and adequacy). Overall, provider shortages were highest for renal pathologists, vascular access coordinators, and dietitians (with 86, 81, and 78% of countries reporting a shortage, respectively), and the shortages were more common in low-income countries. Few countries (35%) reported a shortage in laboratory technicians. This information highlights significant inter- and intra-regional variability in the current capacity for kidney care across the world. Important gaps in awareness, services, workforce, and capacity for optimal care delivery were identified in many countries and regions (4). The findings have implications for policy development with regard to establishment of robust kidney care programs, particularly for low-income and lower-middle-income countries (46). The Global Kidney Health Atlas has therefore provided a baseline understanding of where countries and regions stand with respect to several domains of the health system, thus allowing the monitoring of progress through the implementation of various strategies aimed at achieving equitable and quality care for the many patients with kidney disease across the globe.

How could this information be used to mitigate existing barriers to kidney care? First, basic infrastructure for services must be strengthened at the primary care level for early detection and management of AKI and CKD across all countries (46). Second, although optimal kidney care obviously should emphasize prevention to reduce adverse consequences of kidney disease at the population level, countries (particularly low-income and lower-middle-income countries) should be supported at the same time to adopt more pragmatic approaches in providing RRT. For example, acute peritoneal dialysis could be an attractive modality for AKI, because this type of dialysis is as effective as hemodialysis, requires far less infrastructure, and can be performed with solutions and catheters adapted to local resources (47). Third, kidney transplantation should be encouraged through increased awareness among the public and political leaders across countries, because this is the clinically optimal modality of RRT and it is also cost-effective, provided that costs of the surgery and long-term medication and follow-up are made sustainable through public (and/or private) funding (48). Currently, most kidney transplants are conducted in high-income countries in part because of lack of resources and knowledge in low-income and lower-middle-income countries, as well as cultural practices and absence of legal frameworks governing organ donation (48).

## Conclusion

Socially disadvantaged persons experience a disproportionate burden of kidney disease worldwide. The provision and delivery of kidney care varies widely across the world. Achieving universal health coverage worldwide by 2030 is one of the WHO Sustainable Development Goals. Although universal health coverage may not include all elements of kidney care in all countries (because this is usually a function of political, economic, and cultural factors), understanding what is feasible and important for a country or region with a focus on reducing the burden and consequences of kidney disease would be an important step toward achieving kidney health equity.
